# Preserving extracellular vesicles for biomedical applications: consideration of storage stability before and after isolation

**DOI:** 10.1080/10717544.2021.1951896

**Published:** 2021-07-14

**Authors:** Fumin Yuan, Ya-Min Li, Zhuhui Wang

**Affiliations:** a Department of Clinical Medicine, Grade 2018, Xiangya School of Medicine of Central South University, Changsha, China; b Clinical Nursing Teaching and Research Section, Second Xiangya Hospital of Central South University, Changsha, China; c Hunan Testing Institute for Medical Devices, Changsha, China

**Keywords:** Extracellular vesicles, exosomes, preservation, storage, nanomedicine

## Abstract

Extracellular vesicles (EVs) are nanovesicles released by various cell types. EVs are known for cell-to-cell communications and have potent biological activities. Despite great progress in recent years for studies exploring the potentials of EVs for early disease detection, therapeutic application and drug delivery, determination of the favorable storage conditions of EVs has been challenging. The understanding of the impact of storage conditions on EVs before and after isolation is still limited. Storage may change the size, number, contents, functions, and behaviors of EVs. Here, we summarized current studies about the stability of EVs in different conditions, focusing on temperatures, durations, and freezing and thawing cycles. –80 °C seems to remain the most favorable condition for storage of biofluids and isolated EVs, while isolated EVs may be stored at 4 °C shortly. Lyophilization is promising for storage of EV products. Challenges remain in the understanding of storage-mediated change in EVs and in the development of advanced preservation techniques of EVs.

## 1. Introduction

Extracellular vesicles (EVs) are cell-derived nanoscale vesicles, known for their key roles in between-cell communications (Tkach & Thery, 2016). Major subtypes of EVs are exosomes, microvesicles, and apoptotic bodies according to their cellular origin. EVs could be released from almost all cell types and exist in various kinds of biofluids, including blood, urine, breast milk, saliva, and semen (Li et al., 2019). A growing number of studies have demonstrated the mediating role of EVs in disease development and progression such as malignant tumors (Wu et al., 2019), cardiovascular diseases (Jansen et al., 2017), metabolic disorders (Huang-Doran et al., 2017), and neurodegenerative diseases (Matsumoto et al., 2017). EVs have shown the potential in diagnosis and therapy for clinical application and are emerging as attractive theranostic tools for their contents, including proteins, lipids, and nucleic acids, generated from their parent cells (Pathan et al., 2019).

EVs are obtained from biofluids or supernatant of cell culture media. For the collection of EVs from biofluids, samples were often obtained from the biobank; for the collection of EVs from the supernatant of cell culture media, large-scale production is not feasible. Hence, samples or isolated EVs are often stored before analysis and application (Kusuma et al., 2018). However, little is known regarding how to preserve samples or isolated EVs before functional analysis or therapeutic application. To fully explore the potentials of EVs for their contents and functions, EVs need to be stored and then used or analyzed before alteration. Otherwise, the findings of EV studies may not be reliable due to changes from impacts of storage.

Biofluid samples are often stored according to conventional protocols, but isolated EVs have been recommended to be stored at −80 °C (Witwer et al., 2013; Thery et al., 2018). The influence of different storage temperatures and durations on EVs have been evaluated by a few studies, with inconsistent results. For particle numbers, a study collected exosomes from HEK293 cells and found that the storage at 4 °C or 37 °C for 24 h has higher particle numbers than storage at freezing temperatures (Cheng et al., 2019). However, in another study, it was reported that freezing temperatures preserved most EV particles, and 4 °C and 20 °C would cause significant loss of EV numbers (Lőrincz et al., 2014). For particle size, the increased mean size of exosomes after storage at 4 °C for four days has been observed in a study (Maroto et al., 2017). However, a study also found a decreasing mean size of exosomes over storage time at 4 °C (Sokolova et al., 2011). There were also conflicting results from studies investigating various storage factors, such as temperatures, freezing and thawing cycles, and pH, on different aspects of EVs. Another important factor affecting stability of EVs is the storage buffer, though there seems to an agreement that PBS is suitable for EV storage (Witwer et al., 2013) and have been used in most studies (Kusuma et al., 2018). An emerging technique for preserving EVs is lyophilization (freeze drying), which is a two-step method widely used for preserving thermo-liable materials. Freeze-dried material can be stored and recovered feasibly. However, there is a risk of affecting EV integrity and contents for the lyophilization process and the choice of cryoprotectant should be cautious.

Overall, there is a lack of consensus regarding the influence of preservation method on EVs. Toward further clinical translation of EVs for disease diagnosis and therapy, the understanding of how storage conditions affect EV quality is urgent. In this review, we aim to summarize current studies reporting the influence of storage conditions on EVs, including particle properties, contents, and functions. Here, we focus on storage temperatures and durations, the impact of storage conditions on biofluids before EV collection and on isolated was separately discussed. It is suggested for further EV studies to report their storage strategies for EVs.

## 2. Preservation of biofluids samples before EVs isolation

**Figure 1.**
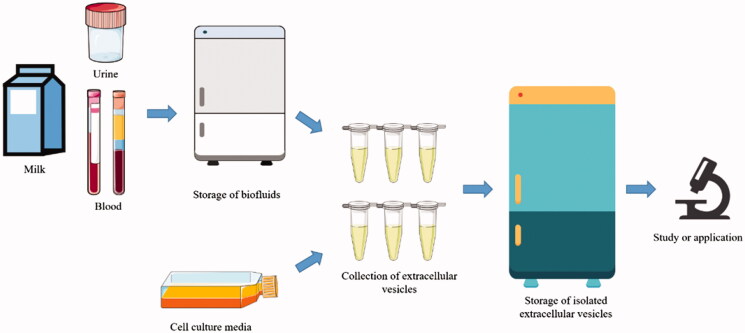
Illustration of the storage of biofluids samples and collection of extracellular vesicles from biofluids or cell culture media before study or application.

As EVs have shown potentials for theranostic applications, the collection of existing samples in the biobank is feasible for the analysis of small EVs (sEVs). Various kinds of biofluids contain EVs. Storage of biofluids seems to be inevitable before isolating EVs as most samples were obtained from the biobank and cannot be processed freshly (Figure 1). Hence, a challenge is that samples in the biobank could have been stored for a long term. In addition, samples in the biobank are often stored in freezers and may expose to freeze–thaw cycles. Determining the impact of storage conditions on samples before EV collection would help researchers to place samples in an optimal environment and utilize them without affecting properties EVs. Studies reporting the influence of storage conditions of different biofluids samples on EVs were summarized (Table 1).

**Table 1.** Summary of studies investigating influence of storage conditions on EVs before isolation.

Biofluid	Type of EV	Isolation	Storage conditions		Changes after storage			Other	Ref.
			Temperature	Duration	Particles	Contents	Functions		
Blood	sEVs	ExoQuick	RT, 4 °C, −20 °C, −40 °C, −80 °C, −160 °C	Days or months	NA	Storage at RT and 4 °C showed higher signal intensities for long-term storage but lower signal intensities for short-term storage	NA	Individual variation may affect the results	Baek et al. (2016)
Plasma	EVs	None	–80 °C; single freezing and thawing	12–20 m	Decreased level of AnnV^+^ EVs; increased AnnV^+^ EVs after single freezing and thawing	NA	NA	–	Ayers et al. (2011)
Platelet	Nanovesicles	Differential UC	–80 °C + DMSO	1 h	Increased nanovesicle number	NA	Higher procoagulant activity	–	Tegegn et al. (2016)
Milk	EVs	Differential UC	4 °C, −80 °C	2–8 weeks	NA	No change in CD63 and CD9 expression	NA	Contamination of EVs from cell death	Zonneveld et al. (2014)
Semen	Exosomes	ExoQuick	–80 °C	2 and 30 years	No change	Long-term freezing decrease protein levels but not total RNA level	Long-term freezing decrease AChE and anti-HIV activities		Welch et al. (2017)
Urine	Exosomes	Differential UC	RT, 4 °C, −80 °C	2 h, 1 day, 1 week	Decreased EV yield over time	NA	NA	Freezing with protease inhibitors help alleviate nanoparticle reduction	Oosthuyzen et al. (2013)
	Exosomes	Differential UC	−20 °C, −80 °C with protease inhibitor	1 week	NA	Freezing decrease in EV-associated protein and −20 °C caused a major loss in exosomes proteins	NA	Extensive vortexing after thawing markedly increased exosome recovery	Zhou et al. (2006)

AnnV: annexin V; NA: not available; sEVs: small extracellular vesicles; UC: ultracentrifugation.

### 2.1. EVs from blood

Blood samples are potent candidates serving as liquid biopsies, EVs could be obtained from the blood, a major type of biofluid. Blood-derived EVs have been widely explored for their diagnostic and therapeutic potentials (Hu et al., 2020; Zhou et al., 2020). Baek et al. investigated the impact of various pre-analytical factors (temperatures) on sEVs collected from human blood (Baek et al., 2016). They reported that room temperature (RT) and 4 °C storage displayed higher signal intensities of sEVs that were positive for EV protein markers (CD9, CD63, and/or CD81) than lower temperatures for long-term storage but lower signal intensities than lower temperatures for short-term storage. Besides, from their data, freeze-thawing of blood samples seemed to have little influence on the recovery of sEVs. However, it should be noted that blood samples from different individuals may have substantial heterogeneity for the contents of sEVs in blood.

Circulating EVs are involved in several disease processes and increase in circulating EV numbers have been observed in pathological process such as thrombotic and atherosclerosis (Agouni et al., 2019; Peng et al., 2020). The measurement of circulating EVs is of diagnostic value for early disease detection. Hence, storing blood samples for EV detection and analysis is of importance. Ayers et al. used a flow cytometric assay to measure annexin V^+^ (AnnV^+^) EVs from plasma (Ayers et al., 2011). They found that a single freeze–thaw cycle of plasma samples led to an increase in AnnV^+^ EVs. In contrast, long-term storage of plasma samples at −80 °C resulted in decreased EV levels.

Platelet membrane-derived nanovesicles may be important for the procoagulant activity of platelet for transfusion medicine (Helmond et al., 2013). Storage time is critical for the availability of platelet. Currently, platelets are cryopreserved with 6% dimethyl sulfoxide (DMSO) in the clinic for long-term storage for transfusion (Valeri, 1974; Valeri et al., 2005). Preserving the procoagulant activity and desired functions remains a challenge in the development of cryopreservation techniques. Functions of platelet membrane-derived nanovesicles may be affected after cryopreservation. Tegegn et al. reported that the freezing (–80 °C) and thawing process leads to activation of platelets (Tegegn et al., 2016). Due to the membrane transition process, cryopreserved platelets exhibit dramatic changes in membrane integrity which increased the platelet thrombin generation activity.

### 2.2. EVs from milk

Extracellular vesicles in breast milk contain proteins with immunological activities and may play important roles in the development of the neonatal immune system (Adriano et al., 2021). Bovine milk-derived EVs have been developed as vehicles for tumor drug delivery for treatment efficacy and high biocompatibility (Munagala et al., 2016; Agrawal et al., 2017; Somiya et al., 2018). Most studies on human and bovine milk EV have utilized unprocessed milk (frozen or refrigerated) for biomarker profiling, immunological functions analysis, or drug delivery. It has been reported that drug-loaded EVs derived from bovine milk could be stored at –80 °C for nearly a month without alterations in their physical and pharmaceutical properties (Agrawal et al., 2017). –80 °C condition also showed minimal influence on the activity of milk-derived EVs (Munagala et al., 2016). However, the isolation and storage procedures may affect greatly the recovery of EVs in breast milk. Zonneveld et al. found that while storage of unprocessed breast milk at 4 °C or −80 °C had little influence on the protein markers CD63 and CD9, it would result in the death of cells, possibly for the loss of membrane integrity, in breast milk thus contaminate significantly the naïve EV population in breast milk for further analysis and usage (Zonneveld et al., 2014).

### 2.3. EVs from semen

EVs from semen have been reported to have anti-HIV-1 activity (Welch et al., 2020) and may be used as biomarkers for prostate cancer diagnosis (Barcelo et al., 2019). However, it is not practical to process semen samples immediately upon collection for EVs isolation; therefore, studies investigating the influence of storage conditions on semen samples are required for downstream theranostic use of EVs. To this end, Madison et al. reported EVs from semen have anti-HIV bioactivity and the storage at –80 °C for short term would not impact the EV yield or the bioactivity (Madison et al., 2014, 2015). Further, Welch et al. evaluated the physical properties and bioactivity of EVs from semen after storage for short-term (2 years) or long-term (30 years) freezing at –80 °C (Welch et al., 2017). They found that long-term storage of semen samples at freezing temperatures would not alter EV size or morphology as well as the total content of RNA. But the total protein level and profiles were observed to be decreasing over freezing storage. More importantly, Welch et al. observed a remarkable decreased acetylcholine-esterase (AChE) and anti-HIV-1 activity of EVs obtained from semen after long-term storage (Welch et al., 2017, 2020). Although the small sample size limits conclusions, their data that suggested long-term freezing would damage bioactivity of semen-derived EVs warranted further investigations.

### 2.4. EVs from urine

Urine samples are important source of biomarkers and may be convenient source of EVs. For storage of urine samples before EV collection, Oosthuyzen et al. identified a subpopulation of CD24^+^ and AQP2^+^ particles of exosome size in human urine after storage by NTA and reported that urine stored at RT, 4 °C or freezing temperatures all showed reduced nanoparticle concentration, but freezing at −80 °C with the addition of protease inhibitors produced the least reduction (Oosthuyzen et al., 2013). In another study, Zhou et al. showed that the storage temperature of urine samples is critical for EV quality. Compared to fresh urine, –20 °C storage condition would lead to great loss of EV numbers, but the decrease was not significant as observed for storage at –80 °C. Besides, they found that the loss of EV in frozen urine samples could be saved by vortexing for thawing after −20 °C and −80 °C storage (Zhou et al., 2006). Overall, these studies suggested that EV may degrade soon after urine collection (<2 hours) and the addition of protease inhibitors would help prevent loss of EV numbers for urine samples stored at −80 °C.

## 3. Preservation of isolated EVs

Isolated EVs are often stored in small packages in parts before further analysis and usage (Figure 1). Either for exploring the potential of EVs as biomarkers or engineering EVs as drug delivery systems, the storage of EVs is inevitable. Particle numbers and specific contents in EVs may change or be lost during storage. Therefore, the understanding of the influence of storage conditions on isolated EVs is also important for sufficient reveal theranostic potentials of EVs and repetition. Studies reporting the influence of storage conditions on isolated EVs were summarized (Table 2).

**Table 2.** Summary of studies investigating influence of storage conditions on isolated EVs.

EV source	Isolation	Storage		Changes of EVs after storage			Other	Ref.
		Condition	Duration	Particles	Contents	Functions		
Plasma	ExoQuick	4 °C, −20 °C, −80 °C	2 weeks to 2 years	NA	4 °C for 2 weeks decreased RNA levels; freezing showed no change in RNA or protein levels	NA	–	Ge et al. (2014)
Serum	ExoQuick	RT, 4 °C	6–168 h	NA	RT for 24 h showed no change in CD63, TSG101 and DNA concentration; 4 °C for 1 week showed no change in CD63, TSG101 and DNA concentration	NA	–	Jin et al. (2016)
		Freeze thaws	1, 3, and 5 cycles	NA	No change in CD63, TSG101 but dramatic decrease in DNA concentration	NA	–	
BALF	Differential UC	4 °C, −80 °C	4 days	Increased size and decreased zeta potential	Change in protein composition and level with lower temperature	NA	Distinct loss of protein at different storage temperatures	Maroto et al. (2017)
HEK 293T cells	Differential UC	–20 °C, 4 °C, and 37 °C	0–192 h	Decreased size at 4 °C and 37 °C; multiple freezing and thawing did not affect the exosome size	NA	NA	Multiple UC also did not change the exosome size	Sokolova et al. (2011)
HEK 293T cells	ExoQuick	RT, 4 °C, −20 °C, −70 °C	10 days	NA	RT and 4 °C showed significant decrease in CD63; lower temperature showed less decrease in total protein and RNA	NA	–	Lee et al. (2016)
HEK 293T cells	ExtraPEG	–80 °C, −20 °C, 4 °C, 37 °C, 60 °C at pH 4, 7, or 10	24 h	4 °C showed highest exosome concentration; pH 4 and pH 10 lead to loss of exosomes	4 °C showed the highest level of protein markers ALIX, HSP70, and TSG101; pH 4 and pH 10 lead to loss of exosome	Less cellular uptake at 4 °C storage; storage at pH 4 and pH 10 led to more cellular uptake	–	Cheng et al. (2019)
		Freezing and thawing circles	24 h	Freezing and thawing circles decrease exosome concentration	Freezing and thawing circles decrease protein level	Freezing and thawing circles decrease cellular uptake	–	
PMNs	Differential centrifugation	+20 °C, +4 °C, −20 °C, −80 °C	0–28 days	Storage at +20 °C, or +4 °C showed significant decrease of EV number after 1 day; storage at −20 °C induced a shift in EV size by 28 days	NA	Storage at +20 °C, or +4 °C showed significant decrease of antibacterial effect of EV after 1 day; storage at −20 °C induced complete loss of antibacterial function by 28 days	Common cryoprotectants would induce EV lysis	Lőrincz et al. (2014)
bEnd.3 cells	Differential UC	4 °C, −20 °C, −80 °C	0–28 days	Decreased particle number and widened size range over time for all storage temperatures	Decreased protein and RNA levels over time at 4 °C but not at freezing temperatures	4 °C storage decreased cellular uptake efficiency; 80 °C storage showed stable fluorescence signals in mice and in *ex vivo* organs up to 28 days	–	Wu et al. (2021)
		Freeze thaws	1–5 cycles	Decreased number of sEVs over freezing and thawing circles for all freezing temperatures	NA	NA	–	

NA: not available; PMNs: neutrophilic granulocytes; sEVs: small extracellular vesicles; UC: ultracentrifugation.

### 3.1. Isolated EVs from blood

EVs in blood have been widely explored for the development of biomarkers and diagnostic tools for various diseases (Povero et al., 2020; Yee et al., 2020). EVs isolated from blood are generally stored, at least for a short period, before analysis of their contents of proteins, nucleic acid, etc. To determine the storage stability of circulating miRNA in exosomes isolated from plasma, Ge et al. quantified exosomal miRNA after storage at different temperatures (Ge et al., 2014). They found that exosomal miRNA was stable under freezing conditions (–20 °C and –80 °C) with no significant change up to two years. But short-term (2 weeks) storage at 4 °C would decrease the exosomal RNA level, indicating that exosomal miRNAs from plasma can be stored and used for biomarker research under freezing storage conditions based on their stability. In another study, Jin et al. found that isolated exosomes from serum could only be stored at RT up to 24 h without significant change in CD63, TSG101, and DNA concentration; also, if stored at 4 °C, increased stability of isolated exosomes was observed for no change in CD63, TSG101, and DNA concentration for 1 week (Jin et al., 2016). Besides, they found that the freezing and thawing cycles have little influence on proteins (CD63 and TSG101) markers but decreased dramatically the DNA concentration in exosomes.

### 3.2. Isolated EVs from broncheoalveolar lavage fluid (BALF)

Airway EVs have shown therapeutic potentials for pulmonary diseases, such as asthma and chronic obstructive pulmonary disease (Takahashi & Kubo, 2014; Wahlund et al., 2017). Airway EVs can be obtained from BALF, but the storage condition may affect the integrity and contents of airway EVs. Maroto et al. stored exosomes isolated from BALF at 4 °C, –80 °C for four days and then evaluated the particle morphology and protein profiles (Maroto et al., 2017). They reported that storage conditions have major impacts on the characteristics, morphology, and protein profiles of BALF-derived EVs. More importantly, they observed loss of distinct protein populations for short-term storage at 4 °C and at –80 °C. It was assumed that the results may attribute to the dissociation of proteins in EV membranes, rather than internal proteins (Maroto et al., 2017). Overall, this study highlighted the storage-induced damage to EVs.

### 3.3. Isolated EVs from the supernatant of cell culture media

In addition to biofluids, mounting numbers of EV-related studies obtained EVs from the supernatant of cell culture media (Patel et al., 2017; Yang et al., 2020). Unlike biofluids, the supernatant of cell culture media is ready for EVs isolation by centrifugation (Livshits et al., 2015) or other methods (Ayala-Mar et al., 2019). Hence, samples were generally processed without delay and EV-related studies were interested in the impact of storage conditions on isolated EVs instead of raw supernatant of cell culture media. For investigating the influence of storage conditions on EVs isolated from the supernatant of cell culture media, most studies used human embryonic kidney 293 cells (HEK 293) s for EV production. Sokolova et al. followed the size of exosomes derived from HEK 293T cells during storage at 37 °C, 4 °C, and −20 °C (including freezing and thawing) by NTA (Sokolova et al., 2011). They found the decreased size of exosomes overtime during storage at 37 °C and 4 °C, but multiple freezing and thawing did not affect the exosome size. For contents in EVs, Lee et al. investigated the change to protein and RNA contents of HEK 293T cell-derived exosomes after storage at various temperatures for 10 days (Lee et al., 2016). They found that the storage at RT and 4 °C decreased significantly protein CD63 but not CD9 level in isolated exosomes. Freezing conditions reduced the loss of protein and RNA in exosomes. In another study, Cheng et al. investigated the impact of a variety of storage factors (temperature, cycles of freezing and thawing, pH) on exosomes in regards to the quantity and cellular uptake behavior (Cheng et al., 2019). They found that, for short-term storage (24 h), 4 °C had the highest concentration of exosomes as well as exosomal markers ALIX, HSP70, and TSG101. Freezing and thawing decreased exosome concentration as well as protein level over cycles. Acid or alkaline environments were not suitable for exosome preservation. Freezing and thawing cycles decreased significantly the quality of exosomes. However, in contrast to the result of characterization, their cellular uptake study demonstrated higher cellular uptake of single exosomes after storage at −80 °C, −20 °C, 37 °C, and 60 °C than at 4 °C, the storage at pH 4 and pH 10 also surprisingly led to more uptake of exosomes by cells than at pH 7.

EVs derived from other cells have also been evaluated for storage stability. Lőrincz et al. studied the effect of different storage conditions on both physical and functional properties of neutrophilic granulocytes (PMN)-derived EVs (Lőrincz et al., 2014). They provided convincing data from flow cytometry that the storage at 4 °C for a day would lead to a significant decrease of EV quantity and antibacterial activity. When frozen at –20 °C, EV could be preserved without the decrease in numbers, but a shift in size and substantial loss of antibacterial activity was observed after 28 days of storage. Further, frozen at –80 °C preserved both physical and functional properties of EVs at 28 days. Hence, the authors recommended to kept EVs at –80 °C, preferably not longer than seven days, if storage is needed. For functional tests, it was recommended to use freshly isolated EVs without long-term storage or freezing.

Similar findings were reported in a recent study by Wu et al. which comprehensively evaluated the influence of storage temperatures (4 °C, −20 °C, and −80 °C) and durations on particle, contents, and behavior of bEnd.3 cells-derived sEVs (Wu et al., 2021). bEnd.3 cell was selected for sEVs production for their potential brain targeting effects to observing the impact of storage conditions on their biodistribution. For sEVs particle characterization, the number of exosome-size particles was decreasing while the size range was increasing during storage for all temperatures. For cellular uptake of sEVs, storage at 4 °C significantly decreased the autologous cellular uptake efficiency. Also, a decreasing uptake efficiency over time was observed for sEVs stored at −20 °C. However, a comparable uptake efficiency to fresh sEVs was observed for sEVs stored at −80 °C within three weeks. Further, in their biodistribution study, similar findings were observed. Freshly isolated sEVs showed the strongest signals in the whole body, as well as in the brain. Significantly decreased signals were observed for sEVs storage at 4 °C or −20 °C, and the signal can hardly be detected in the brains. However, sEVs stored at −80 °C showed stable biodistribution signals within 14 days of storage, despite decreased signals in brains after 14 days of storage. The authors recommend storing isolated sEVs at −80 °C to preserve their contents and functions.

## 4. Determine the optimal storage condition for EVs

From current studies, storage conditions impact substantially the yield, integrity, quantity, contents, and functions of EVs. For biofluids, most samples were stored at –80 °C. While freezing at –80 °C may decrease the number of EV particles over time, storage at higher temperatures would cause more significant loss. Besides, most studies found that freezing temperature would not alter the contents in EVs for long-term storage (Jeyaram & Jay, 2017). Therefore, for collecting EVs from biofluids, samples could be stored at −80 °C. However, for isolated EVs, the storage conditions are depending on the study design and purpose. Major factors, including storage temperature, storage duration, and freezing and thawing cycles, of EV preservation are discussed in the following sections.

### 4.1. Storage temperature

A number of studies have evaluated the effect of storage temperatures on EVs. Despite inconsistent results, most studies reported that 4 °C, −20 °C, or −80 °C conditions are acceptable for EV storage. However, determining the most favorable storage conditions for EVs is dependent on the study purpose and storage duration. For short-term storage of days or several weeks, isolated EVs could be stored at 4 °C. For long-term storage of years, isolated EVs are recommended to be stored at −80 °C.

### 4.2. Freezing and thawing cycles

While there has been a study reporting stability of EVs after freezing and thawing cycles, the freezing and thawing cycle was on biofluids samples instead of isolated EVs. Isolated EVs may be more structurally fragile when suspended in solutions such as phosphate-buffered saline than in biofluids. Besides, there are also studies that showed that EVs are structurally susceptible to repeated freeze–thaw cycles for their vulnerable phosphatidylserine. Therefore, it would be wise to prevent freezing and thawing cycles when storing EVs. For storage of isolated EVs at freezing temperatures, additional freezing and thawing cycles should be avoided as it would damage the integrity of EVs seriously.

### 4.3. Storage duration

In line with storage temperatures, the storage duration of isolated EVs is dependent on the study design and purpose. Generally, for short-term storage, 4 °C is preferred as it can avoid freezing and thawing cycles. While there has been a lack of continuous follow-up study for the impact of storage conditions on isolated EVs for long-term (years), −80 °C is recommended. Further investigations of the long-term effect of storage conditions on isolated EVs are encouraged.

## 5. Challenges and future perspective

Storage of EVs is the last step before analysis or functional studies. For successful translation of EVs for clinical applications, there are various processes before storage. Whether for diagnostic or therapeutic purposes, the combined effort of EV production, isolation, purification, preparation, characterization, storage, transport, and quality control is required to develop a protocol for EV study. For the therapeutic application of EVs, the supply chain is of importance for maintaining EV quality; therefore, determine the optimal storage condition for EVs is necessary for retaining the functions of EVs and maximizing the therapeutic effects.

EVs are heterologous populations, current studies reporting the influence of storage conditions on EVs used EVs obtained from different sources. More importantly, the isolation method may affect the results as different isolation methods may result in different EV subtypes. A number of studies used commercial kits to isolate EVs. These kits generally have limited reproducibility for the lack of technical and material details. Therefore, it should be cautious when referring to the conclusions in previous reports. In addition, previous studies were focused on several aspects of EVs, such as particles, morphology, contents, or functions. This provided limited information on EVs after storage and can hardly reflect the whole property of EVs.

There are other emerging techniques for EV storage such as lyophilization (Charoenviriyakul et al., 2018; Bari et al., 2019). The lyophilization has been used for the preservation of materials that are thermolabile (Assegehegn et al., 2020). Charoenviriyakul et al. reported that lyophilization of isolated exosomes, with trehalose as cryoprotectant, prevented the aggregation of exosomes for storage at RT. Also, recovered exosomes exhibited robust pharmacokinetic profiles in mice (Charoenviriyakul et al., 2018). In another study, mannitol was used as cryoprotectant for protecting exosomes during lyophilization (Bari et al., 2019). Freeze-dried exosomes were stored at −20 °C and were ready-to-use. Overall, the lyophilization technique has been considered as a cost-saving strategy for EV preservation and may be used to extend the shelf life of EVs without affecting their particle morphology and contents when stored at RT (Noguchi et al., 2019; Liu et al., 2021). Freeze-drying technique holds great promise for translation of EV into therapeutic products. However, the studies reporting the lyophilization technique of EVs are preliminary and lack standard protocols. Besides, the influence of cryoprotectants on sEVs preservation requires further investigation.

## 6. Conclusions

The storage conditions have major impacts on EVs, determining the optimal storage condition is critical for EV integrity and functions. From current evidence, it seems that –80 °C would be the most favorable condition for the storage of biofluids and isolated EVs. However, −80 °C storage has limitations such as high cost and transportation. For short-term storage of EVs, such as for bench works, EVs may be stored at 4 °C. The understanding of how storage affects EV particle, function, and activity is worth further investigation for improving the storage strategy and the development of cyropreservation method of EVs. Also, further advanced preservation techniques of EVs with high viability and low cost are encouraged.
